# Appendicitis Complicating Pregnancy

**Published:** 1917-03

**Authors:** Aime’ Paul Heineck

**Affiliations:** Professor of Surgery, Chicago College of Medicine and Surgery Chicago


### APPENDICITIS COMPLICATING PREGNANCY.

**By**

**Aime' Paul Heineck, M. D.**

**Professor of Surgery, Chicago College of Medicine and Surgery Chicago.**

(Continued)

### Indications for Operation.

Clinical cures obtained by medicinal measures are rarely anatomical cures. Starvation treatment is debilitating to the mother, is unfavorable to fetal growth. Perforation, abscess, general peritonitis, subdiaphragmatic abscess, thrombosis and embolism are possible results of expectant treatment. Better to remove too many appendices than too few. Be not deterred by the possibility of a difficult operation for the results of early operation are satisfactory and the mortality low.

Operate early in the attack and early in the course of pregnancy. As a general proposition, operation does not interrupt pregnancy. The triumphs of ovariotomy and hysterectomy in pregnancy are well known; in appendicitis operation is even more urgent. Accumulated instances are on record in which pregnant uteri have been operated upon, cauterized, etc., in which ovarian and other pelvic tumors have been removed without pregnancy being terminated. The high mortality of appendicitis in pregnant women is due to fatal temporization. Placental, uterine and peritoneal infections are such serious complications that one should, if possible, operate before the inflammatory process has extended beyond the appendical wall, before abscess formation has taken place, before the onset of peritoneal or other complications.

Operate early in gestation. At that period the uterus is not large enough to be in the way. The operation is less difficult; the tendency to the interruption of pregnancy is minimal and the percentage of maternal recoveries is higher. The danger of recurrence in the latter months of gestation calls for operation during the attack; if that be not feasible an interval operation should be performed as long before the labor as possible.

Operation in fifty cases of non-perforative appendicitis gave only one maternal death and seven abortions. In fifty-five cases of diffuse peritonitis secondary to appendicitis, there were forty-four maternal deaths, only one child was saved, all the others were born prematurely or died soon after birth from weakness, or the illness of the mother resulted fatally before the termination of labor.

### Treatment.

Interruption of pregnancy is not indicated; it increases the danger. Rest should be enjoined; during the operation, the uterus should be handled and exposed as little as possible; after the operation, opiates should be administered. In a clean case, the operative manipulation is slight. Artificial evacuation of the uterus before laparotomy is indicated only when the child is dead or when there are appreciable signs of labor. If the uterus be artificially emptied before the seventh month, the child will be definitely lost and the patient not improved. By evacutaing an appendiceal abscess before emptying the uterus one avoids flooding the free peritoneal cavity with pus. Operations for appendicitis are performed under local or general anaesthesia. Some operators resort to lumbar anaesthesia. Operate as rapidly as is consistent with thoroughness and the patient's welfare.

The operation of election is appendectomy, the technique of which is little influenced by the presence of pregnancy. The same surgical principles are applicable in the pregnant as in the non-pregnant.

When in doubt as to whether the case is one of appendicitis, salpingitis, tubal pregnancy or other pathological conditions, use a supra-pubic median incision. This incision affords easy access to most of the pelvic contents and though it is not the incision of election for exposure of the appendix, it is a very serviceable incision. In cases of combined appendicitis and salpingitis, combined appendicitis and tubal pregnancy, combined uterine myoma and appendicitis, etc., the median infraumbilical incision should be employed.

In 125 of our cases the appendix was removed. In forty-three cases, it is not stated whether it was removed or not. In five cases, it was sought but not found, and therefore, not removed. Each of these cases presented an abscess, which was evacuated and drained. If the appendix be imbedded in a mass of firm inflammatory adhesions, it can be removed by shelling it out of its peritoneal coat.

An appendiceal abscess should be opened at its point of maximal bulging; preferably through a cutaneous surface. If the appendix be not easily found, be content with incising the abscess, evacuating its contents and resorting to tube or gauze drainage. A subsequent operation will rarely be required to remove the appendix. Appendiceal abscesses have been opened and drained through the vagina. Appendiceal abscesses have also been opened through the rectum. These are exceptional procedures; methods of necessity, not of election.

The post operative treatment is that which is employed in the non-gravid modified only by a longer sojourn in bed, thereby giving time for firm consolidation of the operative wound.

**Post Operative Complications and Sequelae.**

In cases of such widely different nature as those herein studied, operated in different surroundings and by different operators, one is not surprised to find noted the occurrence of post operative complications and post operative sequelae. The danger of hernia development after timely operations for appendicitis is practically nil. The protection of the operative scar by the aid of adhesive plaster has been recommended. See that labor be not unduly prolonged.

Among the sequelae reported in these cases were four ventral hernias, three cases of diffuse peritonitis, thrombosis of femoral veins, phlebitis, subphrenic abscess, intestinal fistulae, etc.

**Summary.**

1. Appendicitis occurs at all ages and in both sexes. It presents to all medical men important diagnostic, prognostic and therapeutic features.

2. Appendicitis acute, or chronic, initial, relapsing or recurrent, primary or secondary, complicates pregnancy with greater frequency than is believed. It is the most important complication of pregnancy.

3. It occurs in single and twin gestations; in first, early and late pregnancies; in primiparae, deutiparae, and multiparae.

4. It occurs at all periods of the child-bearing age and at all periods of gestation. It complicates both intra- and extra-uterine pregnancies and can coexist with other disease processes to which it may be primary, secondary or coincidental.

5. Gestation exerts no untoward influence upon the normal appendix. It can and frequently does aggravate existing, or determine new inflammatory disturbances in appendices deviating from the normal in form, length, mobility, location, etc., in appendices bound down by adhesions or the seat of inflammatory or other degenerative changes. Pregnancy does not relieve the dangers of appendicitis, but aggravates them.

6. Appendicitis and uni or bilateral tubal pregnancy are frequently mistaken for each other. They may occur simultaneously or consecutively, may be either primary or secandary to, or independent of each other.

7. In appendicitis, in ectopic pregnancy and in combined appendicitis and ectopic pregnancy, of obscure symptomatology, it matters not whether you are certain or in doubt as to the real diagnosis, early and timely operative treatment is imperatively indicated.

8. During gestation, every type of appendicitis may occur: Adhesive, catarrhal, gangrenous, ulcerative, obliterative, perforative and suppurative.

9. Appendicitis with adhesion formation is of great significance because adhesions of inflammatory origin can, (a) incarcerate the pregnant uterus in the pelvis and mechanically hinder the enlargement of the uterus, (b) impair the contractibility of the uterus, (c) interfere with uterine labor contractions, (d) entail subinvolution, (e) induce sterility, (f) disturb tubal and ovarian integrity of function and of structure, (g) determine ileus, (h) produce abortion, and, (i) lead to extra-uterine pregnancy.

10. Chief among the coexisting pathological conditions noted in appendicitis are simultaneous or consecutive inflammation of the uterus, tubes or other pelvic organs. The close anatomical relations existing between the appendix and the pelvic organs explain their frequent association in disease processes.

11. Appendicitis has a greater morbidity and a higher mortality in the pregnant than in the non-pregnant, operated or non-operated. It may terminate pregnancy.

12. The symptomatology of appendicitis in the pregnant is the same as in the non-pregnant. The clinical picture, however, is blurred by the coexisting symptoms of pregnancy. Diagnostic mistakes may be lessened by keeping in mind that appendicitis occurs in pregnant women; that a history of previous attacks during the same or previous pregnancies can frequently be elicited by thorough and deliberate physical examination. With care, one can in these cases almost always arrive at a correct diagnosis.

13. To establish with certainty the diagnosis of appendicitis during pregnancy, it is necessary to exclude the presence of myalgia due to stretching of abdominal muscles, typhoid fever, ruptured or non-ruptured tubal pregnancy, cholecystitis, salpingitis, ovaritis, adnexitis, ovarian cyst with or without a twisted pedicle, rightsided pyelitis and ureteritis, fecal impaction, hepatic and nephritic colic. At times, any of the forementioned conditions so closely resemble appendicitis as to cause diagnostic errors and operative mistakes.

14. The morbidity and mortality of appendicitis complicating pregnancy and the puerperium are the morbidity and mortality of delay in applying efficient surgical treatment The initial symptoms of the attack do not enable the clinician to foretell accurately how a given case will terminate. What is going to happen in ten, twenty or forty hours following the onset of appendicitis cannot be foreseen. When the condition is dagnosed and remedied early, the mortality is practically nil. Abscess formation may be forestalled by early diagnosis and early operation. The high mortality is due to late diagnosis and late operation. The pregnant woman whose metabolism is good is a good subject for operative measures.

15. Prognosis is better for the mother if there be no interruption of pregnancy spontaneous and otherwise. The bad attacks cause abortions and abortion aggravates the illness.

In the great majority of surgically treated cases there is no interruption of pregnancy and when it does occur it is not due directly to the operation. The interruption of pregnancy is not indicated. It aggravates the prognosis. The fetal prognosis is good in early operated cases.

16. The following prophylactic measures are sound and safe and are recommended for general adoption: (a) During the child-bearing age, recurrent attacks of pelvic pain, dysmenorrhea, menstrual and other pelvic disturbances unassociated with objective pelvic findings are not infrequently due previous attacks. (c) In gravid women, all attacks of indigesence of this etiological factor, the ablation of the appendix is indicated. (b) In laparotomies for conditions other than appendicitis, the appendix should be examined. Should it present any deviation from the normal, its removal is indicated. (c) During the child-bearing age, any woman who has had one or more attacks of appendicitis treated non-operatively should have her appendix removed so as to correct existing pathological conditions and prevent future attacks of appendicitis and complications incident thereto. True prophylaxis in a woman of child-bearing age who has had one or more well marked attacks of appendicitis is an interval operation· It goes without saying that constipation is to be avoided and that other hygenic precautions are to be observed.

17. A definite and accurate diagnosis of acute, chronic or recurrent appendicitis, irrespective of the stage of pregnancy, invariably calls for operation. The disease during pregnancy runs such a rapid destructive course that delay is hazardous Operation should be early and immediate. A case may be rendered hopeless by hesitation and inaction. Temporizing methods are extremely dangerous.

18. Treat appendicitis in the pregnant female as you treat it in the non-pregnant. Every pregnant woman who is a subject of appendicitis should be operated on just as soon as the diagnosis is made, whether the attack is the first, second or third.

The unusual risks of leaving a diseased appendix in the abdominal cavity are much increased by the pregnant state and the evil consequences of another attack, i. e., gangrene or perforation will be correspondingly greater. The danger of recurrence in the later months of pregnancy and in the childbed period calls for operation preferably during the attack. If the patient is not seen in time, one will do the next best thing, an interval operation during the pregnancy. Pregnancy is an additional indication for operation in cases of appendicitis.

19. In inflammatory disease of the appendix, the ideal operation is an appendectomy. In some cases, however, one has to be content with incision, evacuation and drainage of an appendiceal abscess. Exceptionally drainage of abscesses in Douglas' pouch may be effected through the vagina or rectum. Pus should be evacuated irrespective of uterine contents, and irrespective of its location·

20. It is well to keep in mind that for an appendectomy the median incision is contraindicated in the later months of pregnancy, that it is best to avoid or to reduce to a minimum the manipulations of the uterus; opiates are indicated in the after treatment. Labor when it occurs shortly after a laparotomy is not to be unduly prolonged; it may have to be assisted.

